# Silicon saw-tooth refractive lens for high-energy X-rays made using a diamond saw

**DOI:** 10.1107/S0909049510003584

**Published:** 2010-03-17

**Authors:** A. H. Said, S. D. Shastri

**Affiliations:** a Advanced Photon Source, Argonne National Laboratory, Argonne, IL, USA

**Keywords:** saw-tooth lenses, refractive lenses, high-energy X-rays, X-ray optics

## Abstract

Silicon is a material well suited for refractive lenses operating at high X-ray energies (>50 keV), particularly if implemented in a single-crystal form to minimize small-angle scattering. A single-crystal silicon saw-tooth refractive lens, fabricated by a dicing process using a thin diamond wheel, was tested with 115 keV X-rays, giving an ideal 17 µm line focus width in a long focal length, 2:1 ratio demagnification geometry, with a source-to-focus distance of 58.5 m. The fabrication is simple, using resources typically available at any synchrotron facility’s optics shop.

## Abstract 2

A Si saw-tooth refractive lens, fabricated by a dicing process, is demonstrated to focus a 115 keV X-ray beam.

## 1. Introduction

**Figure 1:**
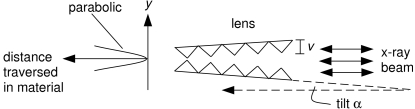
Two opposite-facing saw-tooth structures, tilted symmetrically about the beam axis, impose a parabolic thickness profile. Either piece’s spatial acceptance is at most the tooth height *v*, occurring when the saw-tooth pattern is long enough (>*v*/sinα) for the grazing incidence setting. The beam can enter from either end, leaving the operation unaffected.

For over a decade, refractive lenses have had a significant role in optics for synchrotron radiation sources, during which they have been physically implemented by various means, some conceptually operating through a combination of refractive and diffractive principles. The prevalent scheme, referred to as the compound refractive lens, is based on passing the X-ray beam through a sequential array of ideally parabolic bi-concave walls of a material, thereby imparting convergence (*e.g.* focusing) since material refractive indices for X-rays are less than unity (Tomie, 1994 ▶; Snigirev *et al.*, 1996 ▶; Lengeler *et al.*, 1999 ▶). This communication pertains to a different type of refractive lens, namely the saw-tooth lens, which operates on the principle that a triangular saw-tooth structure, in an overall grazing incidence setting with respect to a beam, presents a parabolic thickness profile, as required for aberration-free refractive optics (Cederström *et al.*, 2000 ▶, 2002 ▶; Dufresne *et al.*, 2001 ▶). A full symmetric parabolic profile requires placement of two such saw-tooth structures face-to-face, canted symmetrically about the beam axis (Fig. 1 ▶). If one constructs a ray directed along the symmetry axis and then continuously translates it away (off-axis in *y*), not only do additional teeth periodically enter into the ray path but each previously entered triangular tooth continues to contribute a linearly increasing thickness against the ray as the displacement progresses. The result is a quadratic arithmetic sum growth in the total thickness traversed as a function of *y*, representing a parabolic profile, approximated in a very fine, piecewise-linear, but continuous fashion. In addition to this desired figure, such a device has other advantages. It has good transmission because the saw-tooth arrangement has no on-axis thickness (*i.e.* unity on-axis transmission). The focal length of such a lens is also easily tuned by symmetric adjustment of the taper angles of the two pieces, which alters the extreme curvature radius *R* of the parabola, through the relation *R* = *v*sinα, where *v* is the tooth height and α is the taper angle with respect to the beam. The focal length is then given by *f* = *R*/δ = (*v*sinα)/δ, where δ = 1 − *n* quantifies the decrement of the material’s refractive index from unity. Additionally, the choice of a single-crystal material for the saw-tooth structure adds the benefit of reducing small-angle scattering halos surrounding focal spots. However, this lens achieves focusing or collimation in one direction only, unless another orthogonally oriented device/set is also present.

A previous article (Shastri *et al.*, 2007 ▶) reported on the successful use of Si saw-tooth lenses at high energies (50–100 keV) for routine focusing (1–20 µm FWHM) and collimation optics applications at the 1-ID undulator beamline at the Advanced Photon Source (APS). Those lenses were fabricated by a process that involved subjecting single-crystal Si to a crystallographically anisotropic chemical etching process (Ribbing *et al.*, 2003 ▶), yielding isosceles teeth of 100–200 µm height, depending on the device. This communication describes a different method of fabricating a Si saw-tooth lens using a thin diamond saw blade. Although the anisotropic etching and other microfabrication approaches (*e.g.* reactive ion etching) are more suitable for smaller structures of high quality, the method described here is simple, requires tools generally accessible at any synchrotron facility, and suffices for long-focal-length (low demagnification) geometries.

This work, including the device specifically described here, was motivated by the desire to upgrade the 115 keV fixed-energy APS beamline 11-ID-C, by incorporating focusing to enable various experiments to be conducted (*e.g.* small samples under high pressure) at a high energy, requiring a beam size of ∼20 µm. Pre-existing physical constraints were that of the end-station instrument being located at 54 m and a limited space available for focusing optics at 35 m from the source point. These dictated a 1.8:1 ratio demagnification geometry (with *f* = 12.3 m), which, in combination with the APS vertical source size of 2.35σ_*y*_ ≃ 26 µm FWHM, would provide a focal width consistent with the requirement. However, the test detailed below was actually performed at the extremely well characterized beamline 1-ID in a not too different 2.0:1 configuration that was permitted there (lens at 39 m with focus 19.5 m further downstream, giving *f* = 13 m).

## 2. Fabrication

**Figure 2:**
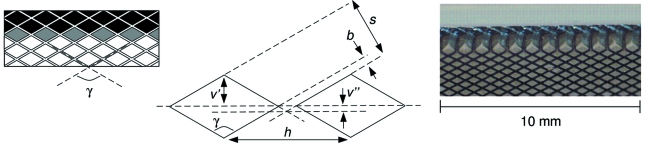
Left: dicing-cut grooves defining parallelogram island pixels. Center: detail of the local saw-tooth profile formed by two corner-adjacent pixels. Right: image of a segment within an actual 130 mm-long device.

**Figure 3:**
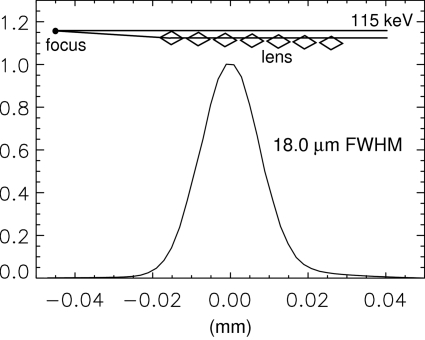
Profile of a 115 keV line focus at 58.5 m from the source created by a single upright lens in a 2:1 demagnification scheme measured with a 5 µm slit. The expected (unconvolved) result for the width is 17.4 µm.

The lens was made by a dicing procedure in Si that is very similar to the method used for many years to produce crystal analyzers for high-resolution inelastic X-ray scattering (Sinn *et al.*, 2002 ▶). A thin diamond wheel cuts two orthogonal sets of parallel equally spaced grooves in a flat Si plate. Since the cut depth is less than the plate thickness, this dicing leaves a two-dimensional array of small square columns (or pixels) protruding from the substrate. Considered here for the lens is the more general possibility in which the two parallel sets of cuts are not necessarily orthogonal but at some arbitrary angle γ, leaving non-rectangular-shaped pixels (Fig. 2 ▶). The overall pattern is oriented within a rectangular plate so that rows of corner-adjacent pixels lie parallel to the plate’s length dimension. After the dicing is complete, under a microscope one selects a long defect-free row of such corner-adjacent pixels (shaded gray) located a few rows inward from the edge and then carefully breaks off all the pixels occupying the intervening rows (shaded black). This allows the chosen row of pixels to reveal, by their exposed column walls, a saw-tooth structure having the slight defect of open valleys arising due to the non-zero cutting width *b* of the blade. Orienting the substrate into the vertical plane parallel to the X-ray beam, with the chosen linear array of pixels slightly tilted from the horizontal, would give vertical focusing (Fig. 3 ▶ inset sketch). Owing to the absence of sharp valleys, the tooth height governing the geometrical acceptance aperture of the lens piece is *v*′, and not the slope-extrapolated tooth height *v*′ + *v*′′. For a complete pair of upright and inverted lens pieces, the aperture would be 2*v*′. However, for the tooth height *v* in the expressions for the parabolic radius *R* and focal length *f* given at the beginning of the previous section, one must use *v*′ + *v*′′ rather than *v*′. In the case of the perfect-valley saw-tooth structure (Fig. 1 ▶), reducing tooth height decreases the aperture, but alleviates the glancing incidence (*i.e.* increases α) at a fixed photon energy and focal length, thereby offering the conveniences of shorter devices. However, the open valleys of the diced lens reduce the aperture, but without changing the glancing angle of incidence.

Based on fabrication experience with typical pixel sizes of ∼1 mm × 1 mm in making Si analyzers, dicing a lens with cut spacing *s* significantly less then 500 µm was deemed undesirable for an initial attempt. A large dicing angle γ reduces the valley defect *v*′′, but also decreases the tooth height *v*′ (*i.e.* the aperture) and lengthens the longitudinal period *h* of the saw-tooth structure (thereby coarsening the quality of the piecewise-linear approximation to the parabolic form). So, in addition to using a nominal 50 µm thin blade that actually cut slightly wider grooves of thickness *b* = 75 µm, the dicing parameters γ = 120° and *s* = 425 µm were chosen. This resulted in a profile with *v*′ = (*s* − *b*)/(2sinγ/2) = 202 µm, *v*′′ = *b*/(2sinγ/2) = 43 µm and *h* = *s*/(cosγ/2) = 850 µm. The cuts were 2 mm deep in a 6 mm-thick Si plate, to give the lens a 2 mm transverse beam acceptance. Refraction of 115 keV X-rays in Si, given by δ = 3.65 × 10^−8^, together with *v*′ + *v*′′ = 245 µm, implies a tilt angle of α = 0.11° to achieve focusing at the *f* = 13 m condition of interest here. In this setting, the fineness along *y* (Fig. 1 ▶) of the piecewise-linear approximation to the ideal parabola is given by the elevation difference between two successive tooth tips, which is *h*sinα = 1.6 µm. For an upright and inverted pair, the full aperture of 2*v*′ ≃ 400 µm would be matched to capture beams from high-energy short-period APS undulators. The minimum length needed for a lens piece to realise the tooth height aperture *v*′ is *v*′/sinα = 105 mm, equivalent to *v*′/(*h*sinα) = 124 teeth participating in the focusing situation relevant here. This requirement was met by the 130 mm-long test pieces diced. The Kulicke and Soffa 984-10 dicing saw was operated with a diamond-coated nickel blade spinning at 15000 r.p.m. and the translation feed of the piece set at 0.25 mm s^−1^. The diced Si was not subjected to any chemical etching process owing to concern about altering the saw-tooth structure.

## 3. Results

Although two diced pieces were tested at beamline 1-ID for vertical focusing, they were characterized one at a time (Fig. 3 ▶ inset sketch), and not as a combined upright/inverted pair (Fig. 1 ▶). Implementing an upright/inverted pair is important for obtaining maximal aperture in eventual application and requires an additional straightforward alignment procedure of steering the two lens pieces’ line foci into coincidence (Shastri *et al.*, 2007 ▶). However, for purposes of assessing the performance of a type of saw-tooth device, a set-up with a single lens piece suffices. The two tested pieces gave almost identical results.

A 115 keV beam was delivered to the lens by a cryogenically cooled, bent double-Laue monochromator (Shastri *et al.*, 2002 ▶) that preserved X-ray divergence and size, leaving the ray propagation effectively unperturbed. White-beam slits defined an aperture giving rise to a 1 mm × 0.2 mm (horizontal × vertical) beam size upon propagation to the location of the lens, exactly matching its vertical acceptance. At the time of this test, a combination of accelerator diagnostics and a separate system of very well characterized X-ray focusing optics indicated Gaussian RMS electron beam source sizes of σ_*x*_ = 270 µm (horizontal) and σ_*y*_ = 11.8 µm (vertical). In addition, this eccentric elliptical source was found to be spatially rotated at a slight angle χ = 1.9°. Such tilts (Dufresne & Khounsary, 2007 ▶) have the important consequence of an amount σ_*x*_sinχ from the large horizontal source size contributing to the vertical source size. For one-dimensional focusing, the transverse inclination of the lens defines the orientation of the line focus. A rotated source combined with a transversely untilted lens produces a line focus that is un­rotated, but broadened. So the effective FWHM vertical source size becomes 2.35[σ_*y*_^2^ + (σ_*x*_sinχ)^2^]^1/2^. Evaluating this and incorporating the 19.5 m/39 m demagnification factor gives an expected 17.4 µm line focus width. Fig. 3 ▶ shows the focal profile measured by vertically scanning 5 µm-wide slits. Considering this slit size, the measured 18.0 µm FWHM size is in agreement with the expected result. Also, the measured width was independent of the scanning aperture’s horizontal extent, which was varied over 0.2–1.0 mm to confirm consistency. The transmission of the Si lens’s effectively parabolic profile as a function of off-axis displacement *y* is Gaussian, decreasing from 100% on-axis (*y* = 0) to 18% at the aperture edge (*y* = 0.2 mm), with an average transmission of 64%. This, along with the acceptance aperture and focal size, would imply a flux density gain consistent with the measured value of about 10, relative to the unfocused flux density of ∼10^12^ photons s^−1^ mm^−2^. Having an upright/inverted lens pair would double the flux density gain (to 20), which would also improve with a smaller vertical source size or no source rotation (*i.e.* χ = 0°).

## 4. Concluding remarks

A simple dicing process on single-crystal Si can produce a saw-tooth refractive lens well suited for focusing high-energy X-rays in a long-focal-length configuration to achieve focal widths going down to 10 µm at 100 keV and 5 µm at 50 keV. Higher demagnifications to obtain smaller beam sizes approaching ∼1 µm require reduced tooth heights to ease challenges associated with device length, glancing incidence angle, and profile errors. For such finer structures more sophisticated microfabrication methods, such as anisotropic or reactive ion etching, are appropriate.

The saw-tooth lens, although fully tunable, is intrinsically a one-dimensionally focusing optic. For two-dimensional focusing, saw-tooth devices refracting in both orthogonal directions must be implemented. In this regard, for focusing in both directions from the same location (*i.e.* with the same demagnification distances), compound refractive lenses with paraboloid elements have an advantage. However, this is not always desired or optimal. Sometimes an experimental technique makes use of a line focus or lenses are needed to collimate the beam in one plane only. Even when a two-dimensional focus is called for, this might be done best from two separate locations. For example, in the long-focal-length vertically focusing geometry presented here, simulations show that there is a slight reduction in flux density gain if one were to focus in both directions from the same location. This is because the potential gain from horizontal focusing is lost by a combination of the weak demagnification of the relatively large horizontal source size and the added horizontal absorption profile of the lens system. Enhanced gains are expected if one were to conduct the vertical and horizontal focusing from different locations, with the latter at higher demagnification, to produce an image spot that is less eccentric (*i.e.* more circular) than the source. One-dimensionally focusing optics are ideal for such arrangements.
